# Functional Cognitive Disorder: diagnostic challenges, clinical features, and future directions in a misunderstood condition

**DOI:** 10.3389/fnagi.2025.1665510

**Published:** 2025-09-16

**Authors:** Ioannis Mavroudis, Foivos Petridis, Katerina Franekova, Malina Visternicu, Viorica Rarinca, Vasile Burlui, Alin Ciobica, Bogdan Novac, Irina Dobrin, Mihai Marian Hogas, Erica Bovari, Cristina Albert, Dimitrios Kazis

**Affiliations:** ^1^Department of Neuroscience, Leeds Teaching Hospitals, NHS Trust, Leeds, United Kingdom; ^2^Third Department of Neurology, Aristotle University of Thessaloniki, Thessaloniki, Greece; ^3^School of Medicine, University of Leeds, Leeds, United Kingdom; ^4^Academy of Romanian Scientists, Bucureti, Romania; ^5^Faculty of Biology, Doctoral School of Biology, “Alexandru Ioan Cuza” University of Iaşi, Iaşi, Romania; ^6^“Ioan Haulica” Institute, Apollonia University, Iaşi, Romania; ^7^Faculty of Geography and Geology, Doctoral School of Geosciences, “Alexandru Ioan Cuza” University of Iaşi, Iaşi, Romania; ^8^Center of Biomedical Research, Romanian Academy, Iaşi, Romania; ^9^Faculty of Medicine, University of Medicine and Pharmacy “Grigore T. Popa”, Iaşi, Romania; ^10^CENEMED Platform for Interdisciplinary Research, “Grigore T. Popa” University of Medicine and Pharmacy of Iasi, Iaşi, Romania; ^11^Preclinical Department, Apollonia University, Iaşi, Romania

**Keywords:** Functional Cognitive Disorder, internal inconsistency, memory complaints, diagnosis, subjective cognitive decline, pseudodementia, metacognition, neuropsychiatry

## Abstract

Functional Cognitive Disorder (FCD), a condition marked by significant subjective cognitive complaints in the absence of identifiable neurological disease, is increasingly recognized as a distinct and underdiagnosed entity in clinical practice. This review synthesizes recent findings to clarify its diagnostic features, differentiate it from other cognitive syndromes such as mild cognitive impairment and dementia, and explore its psychological underpinnings. We examined longitudinal studies, meta-analyses, and clinical frameworks to identify patterns of symptom presentation, cognitive performance, and psychosocial factors. Findings reveal that FCD is characterized by inconsistent cognitive deficits, preserved functional independence, and heightened help-seeking behavior, often accompanied by anxiety, metacognitive dysfunction, and maladaptive beliefs about memory. Unlike neurodegenerative conditions, FCD follows a stable, non-progressive course and shows no evidence of conversion to dementia when accurately diagnosed. Enhanced clinical recognition and structured assessment approaches are crucial for improving diagnostic accuracy, minimizing patient distress, and avoiding unnecessary medical interventions. Further research is needed to standardize diagnostic criteria and develop targeted therapeutic strategies.

## 1 Introduction

Functional Cognitive Disorders are characterized by significant subjective cognitive complaints in the absence of corresponding objective neurological abnormalities typically associated with dementia or other neurodegenerative conditions (Ball et al., 2020; Stone et al., 2015; Marín-Medina et al., 2025; Poole et al., 2019). Increasingly recognized as the cognitive counterpart of FND reflects symptoms that are primarily driven by psychological mechanisms, attentional dysregulation, and impaired metacognition rather than structural brain pathology (Cabreira et al., 2023a; Pennington et al., 2019).

Diagnostic ambiguity and terminological overlap remain common in clinical settings, often leading to confusion among healthcare professionals and distress for patients (Stone et al., 2015; Bailey et al., 2017). Labels such as Subjective Cognitive Decline (SCD), pseudodementia, or the colloquial “worried well” have historically been used to describe individuals presenting with cognitive complaints in the absence of identifiable neurodegenerative processes (Ball et al., 2020; Jessen et al., 2014). However, these terms lack etiological clarity and are frequently unsatisfactory for patients seeking a definitive explanation, often resulting in persistent anxiety and repeated consultations (Rahman-Filipiak et al., 2018).

Clinicians often face considerable challenges in distinguishing FCD from early presentations of MCI or dementia, since the symptoms often appear similar at first glance (Pennington et al., 2015; Cabreira et al., 2023b). Nonetheless, several distinguishing features have been consistently reported in recent research. These include internal inconsistency in cognitive test performance, preserved conversational fluency, a tendency for patients to attend appointments unaccompanied, and the presentation of detailed written notes documenting their cognitive concerns (Reuber et al., 2018; Bharambe and Larner, 2018; McWhirter et al., 2022).

Accurate recognition of FCD is of critical importance (McWhirter et al., 2022). Misdiagnosis may lead to unwarranted investigations, inappropriate treatments, and heightened patient anxiety. In contrast, a correct diagnosis offers reassurance and enables targeted interventions, including psychoeducation, cognitive-behavioral therapy, and metacognitive training, which have shown promise in improving patient outcomes (Teodoro et al., 2018; Bhome et al., 2022).

Despite increasing clinical awareness, there remains a pressing need to standardize diagnostic frameworks, develop validated assessment tools, and promote a positive identification model rather than relying on exclusion (Ball et al., 2020; Cabreira et al., 2023b). This paper aims to clarify the concept of FCD, synthesize practical diagnostic strategies grounded in empirical evidence, and reinforce the view of FCD as a distinct, generally non-progressive clinical entity deserving of focused clinical attention and research.

## 2 Definition and diagnostic challenges

Functional Cognitive Disorder is increasingly recognized as the cognitive counterpart of FND, marked by prominent subjective cognitive complaints without corresponding objective evidence of neurological pathology (Ball et al., 2020; Pennington et al., 2019; Laukaityte and Laukaityte, 2024; Tamilson et al., 2025; Kemp et al., 2022). Unlike neurodegenerative conditions, the symptoms of FCD are believed to arise from psychological mechanisms, attentional dysfunction, and impaired metacognitive monitoring, rather than structural brain damage (Teodoro et al., 2018; Bhome et al., 2019).

A defining diagnostic feature of FCD is internal inconsistency, whereby an individual's cognitive performance fluctuates significantly within the same domain or across tasks, often influenced by situational, emotional, or attentional factors (Ball et al., 2020; Wakefield et al., 2018; Silverberg and Rush, 2024). For example, individuals may demonstrate better delayed recall than immediate recall, or perform inconsistently across similar memory tasks, patterns atypical of progressive neurological diseases.

FCD is frequently misclassified or conflated with related constructs such as SCD, pseudodementia, and the informal label “worried well” (Cabreira et al., 2023a; Jessen et al., 2014; McWhirter et al., 2022). While SCD broadly refers to individuals who report cognitive complaints in the absence of measurable impairment on neuropsychological tests, pseudodementia typically describes cognitive symptoms that emerge secondary to psychiatric disorders, particularly depression, and are considered potentially reversible with adequate treatment (Stone et al., 2015; Rahman-Filipiak et al., 2018). However, these terms lack the specificity and explanatory power offered by the FCD framework, often leaving patients with lingering uncertainty about the cause and significance of their cognitive symptoms (Ball et al., 2020; Bailey et al., 2017).

One of the main challenges for clinicians is the distinguishing FCD from early presentations of MCI or dementia, due to the similarity of reported symptoms and the frequently normal findings on initial cognitive assessments (Cabreira et al., 2023a; Pennington et al., 2015). Despite this overlap, longitudinal studies and structured clinical observations suggest that FCD follows a stable, non-progressive course, distinguishing it from true neurodegenerative conditions (Poole et al., 2019; McWhirter et al., 2022; Schmidtke and Metternich, 2009).

**Figure 1:**
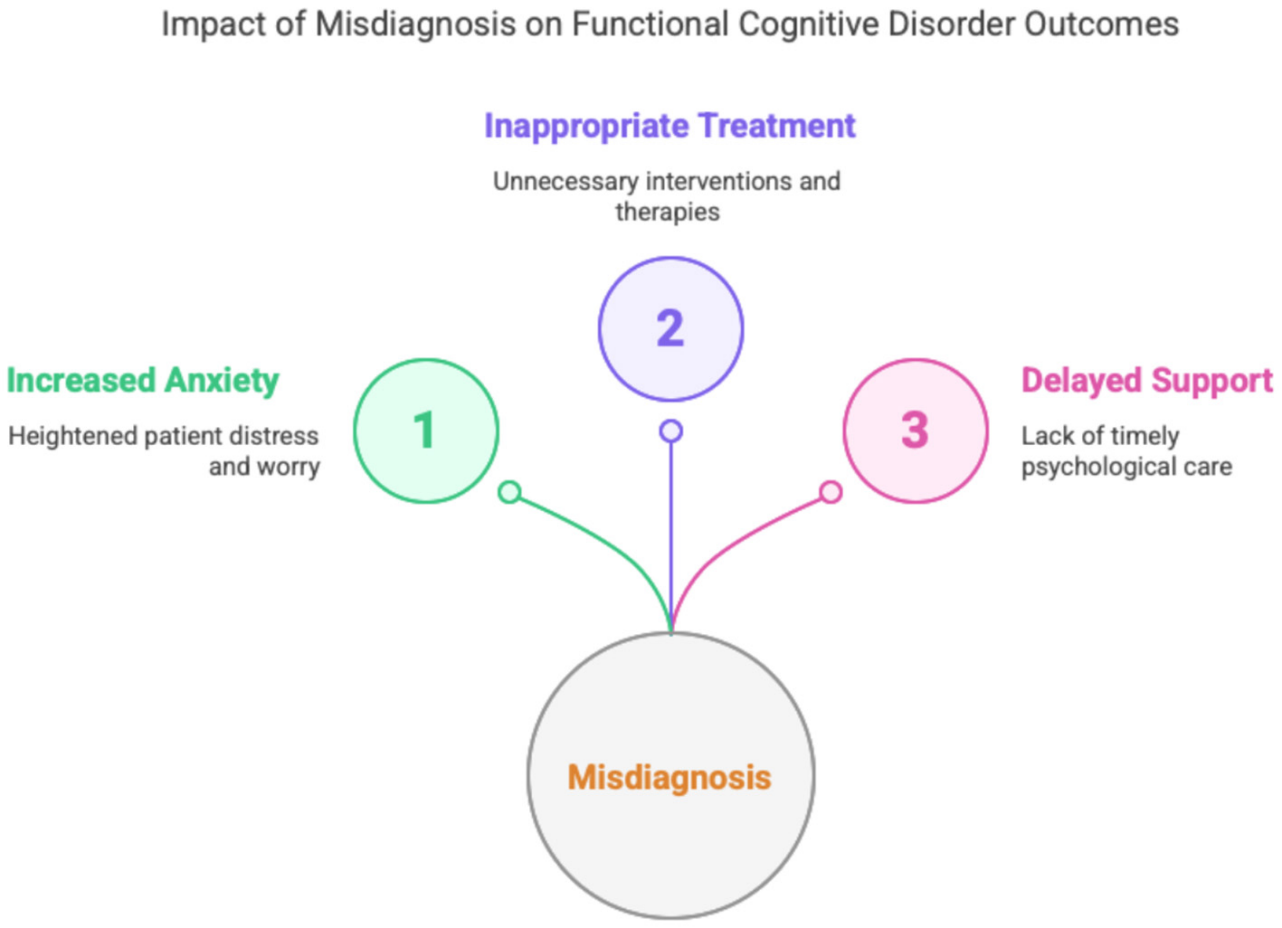
Impact of misdiagnosis on functional cognitive disorder outcomes.

Importantly, misdiagnosis in either direction may have harmful consequences. Patients incorrectly labeled as having MCI can experience increased anxiety about developing dementia, which may further intensify subjective symptoms and negatively affect quality of life (Sabbagh et al., 2020; Edmonds et al., 2014; Voros et al., 2020). In contrast, individuals with undiagnosed FCD may undergo unnecessary investigations, receive inappropriate treatments, or lack access to appropriate psychological support (Ghadiri-Sani and Larner, 2013). This diagram (Figure 1) highlights the three major consequences of misdiagnosing FCD: (1) Increased Anxiety, reflecting the distress experienced by patients when diagnostic uncertainty reinforces fears of neurodegeneration; (2) Inappropriate Treatment, representing the risk of unwarranted pharmacological or therapeutic interventions aimed at misattributed conditions; and (3) Delayed Support, referring to the missed opportunity for timely, targeted psychological care such as cognitive-behavioral therapy or metacognitive rehabilitation. Together, these outcomes underscore the critical importance of early and accurate diagnosis.

In response to these diagnostic complexities, recent expert consensus emphasizes the need for positive diagnostic criteria, including internal inconsistency, characteristic interactional behavior, and communication pattern, rather than relying solely on the exclusion of neurodegenerative disease (Pennington et al., 2019; Reuber et al., 2018). This approach supports clearer clinical decision-making and empowers patients with a coherent, explanatory framework for their symptoms. Once a positive diagnosis of FCD has been established, clinical attention can turn to targeted therapeutic interventions, which are discussed in the following section.

### 2.1. Related concepts and differential diagnosis

FCD shares overlapping features with several cognitive conditions and distinguishing it from these is crucial for accurate diagnosis and effective management. Differential diagnosis requires a careful evaluation of phenomenology, longitudinal course, and psychological context.

#### 2.1.1 Subjective cognitive decline

SCD refers to the perception of cognitive decline in the absence of objective deficits on testing (Jessen et al., 2014). While some cases of SCD precede neurodegenerative disease, others, especially those with internal inconsistency and psychiatric comorbidities, align more closely with FCD (Cabreira et al., 2023a; McWhirter et al., 2020). Unlike FCD, SCD often lacks the prominent metacognitive discrepancies or maladaptive attentional focus seen in functional presentations. A detailed clinical interview can help differentiate between SCD with preclinical Alzheimer's pathology and FCD-related complaints.

#### 2.1.2 Pseudodementia

This older term refers to cognitive symptoms caused by depression or psychiatric disorders. Although pseudodementia can overlap with FCD, the latter is not solely attributable to mood disorders. FCD emphasizes the role of metacognitive dysfunction and cognitive-behavioral mechanisms rather than only affective disturbance (Ball et al., 2020; Pennington et al., 2015). The concept of pseudodementia is falling out of favor due to its oversimplification. Contemporary approaches encourage viewing FCD as a distinct entity with its own cognitive-behavioral profile rather than a subset of depressive symptomatology.

#### 2.1.3 “Worried well”

Often used dismissively, this term refers to cognitively intact individuals with high health anxiety. However, it lacks clinical utility and may invalidate genuine distress (Ball et al., 2020; Bhome et al., 2022). Or example, memory complaints are relatively common among otherwise normal-functioning older adults and may reflect normal aging rather than pathology. Similarly, individuals with depression or anxiety may report cognitive problems that are consistent with their affective disorder or situational stressors, rather than indicating a distinct disorder. In such cases, the degree of complaint is not excessive, daily functioning is generally preserved, and cognitive test performance usually falls within normal limits (Delis and Wetter, 2007). In contrast, individuals with FCD present with disproportionate and persistent cognitive complaints, often accompanied by internal inconsistency in performance, which distinguishes them from the so-called “worried well.” Thus, FCD provides a more respectful and clinically useful framework, allowing clinicians to move beyond reassurance alone and offer targeted interventions, such as psychoeducation or cognitive restructuring, to address maladaptive beliefs and attentional biases (Ball et al., 2020; Bhome et al., 2022; Delis and Wetter, 2007).

#### 2.1.4 Cogniform disorder

A proposed but not widely adopted term, Cogniform Disorder encompasses functional cognitive symptoms not explained by neurological disease. It overlaps conceptually with FCD and reflects ongoing efforts to formalize the diagnosis of functional memory syndromes (Pennington et al., 2015). Cogniform Disorder has been proposed as an analog to somatoform disorders for cognitive complaints. However, FCD offers a more nuanced model grounded in metacognitive theory and empirical findings from functional neuroimaging and behavioral studies.

#### 2.1.5 Functional neurological disorder

FCD is increasingly recognized as the cognitive counterpart to FND. Both share features such as symptom inconsistency, non-progressive course, and associations with psychological distress and attentional dysregulation (Ball et al., 2020; Teodoro et al., 2018). Recognizing this relationship helps align FCD within the broader category of functional disorders. Integrating FCD into the functional disorder spectrum supports unified treatment approaches, such as neuropsychoeducation, attention retraining, and cognitive-behavioral therapy (CBT), across sensory, motor, and cognitive symptom domains. This also encourages multidisciplinary care and avoids fragmented diagnoses.

Although FCD is not formally recognized as a distinct disorder in DSM-5-TR or ICD-11, it conceptually aligns with Functional Neurological Symptom Disorder (Conversion Disorder) in DSM-5-TR and with Dissociative Neurological Symptom Disorder in ICD-11. This nosological linkage highlights the overlap between FCD and FND, particularly regarding symptom inconsistency, non-progressive course, and association with psychological distress. Positioning FCD within this broader spectrum facilitates diagnostic classification, guides management, and helps clinicians direct patients toward evidence-based interventions such as psychoeducation, CBT, or metacognitive training, while avoiding mislabeling as neurodegenerative disease. The lack of a formal diagnostic category for FCD underscores the need for international consensus and standardized clinical guidelines (Butler and Nicholson, 2024; Mavroudis et al., 2025; First et al., 2021).

### 2.2 Management and treatment approaches

Current approaches, including psychoeducation, CBT, and metacognitive training, aim to improve patients' understanding of their cognitive symptoms, reduce maladaptive anxiety, and foster adaptive coping strategies (Bhome et al., 2019). Psychoeducation helps patients recognize the functional nature of their complaints, thereby reducing distress associated with perceived cognitive decline (Bhome et al., 2019; Wolstencroft, 2024). Psychoeducation is often considered the first-line intervention in FCD, helping patients reframe their symptoms as functional rather than neurodegenerative, which reduces health anxiety and catastrophic interpretations (Rueda-Lara et al., 2024). It also provides an explanatory model that normalizes variability and inconsistency in performance. Small observational studies suggest psychoeducation can lower distress and reduce unnecessary healthcare use, but controlled trials are still lacking (Ball et al., 2020; Bhome et al., 2019; Donker et al., 2009).

CBT targets associated maladaptive beliefs, health-related anxiety, and behavioral avoidance, while metacognitive training specifically addresses distortions in self-monitoring, attention regulation, and memory appraisal that often exacerbate subjective cognitive complaints (McWhirter et al., 2020; Day and Thorn, 2022). Pilot studies and case series report improvements in anxiety, subjective cognitive complaints, and quality of life, though objective cognitive gains are less consistent. Evidence is limited by small samples, heterogeneous protocols, and short follow-up (Schmidtke and Metternich, 2009; McWhirter et al., 2020). MCT aims to improve self-monitoring, attention regulation, and memory appraisal, addressing mechanisms believed to sustain FCD (Larner, 2021).

## 3 Clinical signs and diagnostic features

Recognizing FCD in clinical practice hinges on identifying a set of characteristic features that reliably distinguish it from neurodegenerative conditions such as MCI and dementia. One of the most critical of these is internal inconsistency, the observation that a patient's cognitive performance varies significantly across time or contexts within the same cognitive domain, in a manner that is incompatible with structural brain disease and cannot be explained by metabolic changes or other medical conditions (Ball et al., 2020; Pennington et al., 2015). For instance, patients may demonstrate better delayed than immediate recall or maintain fluent conversational abilities while performing poorly on formal memory tasks, suggesting preserved cognitive capacity with context-dependent impairments in retrieval (Cabreira et al., 2023a; Teodoro et al., 2018). This pattern may reflect the influence of cognitive reserve, whereby premorbid intellectual ability, education, or other compensatory mechanisms allow individuals to maintain function despite underlying vulnerabilities (Pappalettera et al., 2024).

A clinically useful operational set of diagnostic criteria for FCD has been proposed by Ball et al. (2020), which includes the following elements:

- One or more symptoms of impaired cognitive function;
- Evidence of functional impact, reflected in avoidance of cognitively demanding tasks of social situation, despite preserved objective performance;
- Evidence of internal inconsistency in performance;
- Symptoms not better explained by another medical, neurological, or psychiatric disorder;
- Symptoms that result in significant distress, impairment, or warrant clinical attention.
- Specify if with/without a linked co-morbidity.

Beyond these core features, systematic reviews and diagnostic meta-analyses have identified additional behavioral and interactional clues that enhance diagnostic confidence. For example, individuals with FCD often present with a distinctive communication profile, characterized by greater fluency and coherence compared to patients with dementia. They frequently provide detailed, unsolicited examples of their cognitive complaints, reflecting preserved episodic memory and meta-awareness (Cabreira et al., 2023a; Reuber et al., 2018).

Interactional behaviors also offer important diagnostic cues (Elsey et al., 2015). Patients with FCD are more likely to attend appointments alone, bring structured written notes about their symptoms, and express high levels of concern, often in contrast to dementia patients, who are commonly accompanied by caregivers who contribute substantively to the clinical narrative (Bharambe and Larner, 2018; McWhirter et al., 2022; Wakefield et al., 2018). One particularly specific marker is the absence of the “head-turning sign,” the behavior of looking toward a caregiver for assistance during memory testing, a behavior frequently seen in dementia but rarely in FCD (Ghadiri-Sani and Larner, 2013; Soysal et al., 2017).

The presence of psychiatric comorbidities, particularly anxiety and depression, is another significant clinical correlate. While FCD is not solely attributable to affective disorders, these comorbidities can contribute to the development and persistence of functional cognitive symptoms (Bhome et al., 2022; McWhirter et al., 2020).

In an observational study, Teodoro et al. (2023) found that patients with FCD spoke for a median of 124 s when describing their memory concerns, markedly longer than the 42-s median in patients with neurodegenerative diagnoses. This verbosity and richness of description suggest preserved verbal fluency and attentional resources, further supporting the functional nature of the disorder.

To enhance diagnostic precision, quantitative tools and structured assessment scales have been developed. The Schmidtke Criteria, in particular, provide a standardized framework with predictive value for non-progression. Other validated questionnaires are detailed in Section 7.4 (Cabreira et al., 2023a; Schmidtke and Metternich, 2009; Mascherek et al., 2011).

Ultimately, the diagnosis of FCD should rest on positive identification of these distinctive features rather than exclusion of organic disease alone (Cabreira et al., 2023b; Van Patten and Bellone, 2023). Careful attention to narrative coherence, metacognitive markers, and psychosocial context allows clinicians to deliver a confident, compassionate diagnosis. This approach not only prevents inappropriate escalation to dementia care pathways but also supports timely psychological intervention and improves overall patient outcomes (Ball et al., 2020; Bhome et al., 2022).

## 4 Communication patterns and interactional profiles

Distinctive communication styles and interpersonal behaviors are central to the clinical recognition of FCD (Pennington et al., 2015). Unlike individuals with neurodegenerative conditions such as Alzheimer's disease, patients with FCD typically exhibit preserved or even enhanced verbal fluency during consultations. These communication features serve as positive diagnostic clues, offering contrast to the vague, hesitant, or under-detailed responses often observed in patients with early dementia (Reuber et al., 2018; Bhome et al., 2022).

A hallmark characteristic of FCD is the patient's ability to provide detailed, unsolicited examples of perceived cognitive failures, such as forgetting appointments or losing track of tasks, which are often described in vivid narrative form (Pennington et al., 2015). This suggests not only intact episodic memory but also heightened meta-awareness of perceived lapses (Teodoro et al., 2023). In contrast, individuals with neurodegenerative conditions frequently rely on generalities, require assistance from informants, or are unable to specify the nature or context of their complaints (Pennington et al., 2015; McWhirter et al., 2020).

Key communication-based features include:

- Longer response duration: In a study by Teodoro et al. (2023), individuals with FCD spoke for a median of 124 s when describing their cognitive concerns, significantly longer than the 42 s observed in patients with neurodegenerative disorders. This verbosity may reflect intact working memory and linguistic fluency, characteristics inconsistent with progressive dementia.
- Higher volume and breadth of complaints: Patients with FCD often report difficulties across multiple cognitive domains, memory, attention, word-finding, multitasking, which may indicate heightened vigilance to normal cognitive fluctuations rather than true multidomain cognitive impairment (Ball et al., 2020; Bhome et al., 2022).
- “Attending alone” behavior: A notable feature in FCD populations is the tendency to arrive unaccompanied at clinical appointments. These patients often bring written summaries or bullet-pointed notes detailing their concerns, behaviors seldom observed in individuals with dementia, who are frequently accompanied by family members who provide collateral information and support (Ball et al., 2020; Reuber et al., 2018).
- Absence of the “head-turning sign”: In neurodegenerative conditions, patients often glance toward caregivers for reassurance or help during cognitive testing, a behavior that is rare in FCD, further reinforcing the functional, rather than organic, nature of their symptoms (Ghadiri-Sani and Larner, 2013).

These interactional features and preserved communication abilities underscore the non-progressive nature of FCD and support a positive diagnostic approach that emphasizes what is present, such as coherence, meta-awareness, and social fluency, rather than focusing solely on what is absent (Reuber et al., 2018; Teodoro et al., 2018).

Recognizing and interpreting these subtle conversational and interpersonal markers requires clinical experience and active listening. However, when used systematically, these features can empower clinicians to move beyond diagnostic exclusion, providing patients with clearer explanations, targeted interventions, and relief from diagnostic uncertainty (Ball et al., 2020; Cabreira et al., 2023b).

## 5 Metacognition and psychological factors

A growing body of research indicates that impaired metacognition and psychological distress are central to the pathophysiology of FCD. Metacognition, the ability to monitor, reflect upon, and evaluate one's cognitive performance, is often dysfunctional in individuals with FCD. This metacognitive dysfunction contributes to increased self-monitoring, hyperawareness, and misinterpretation of normal cognitive fluctuations as pathological decline (Ball et al., 2020; Bhome et al., 2022; McWhirter et al., 2020).

One key psychological trait frequently observed in FCD is memory perfectionism, whereby individuals hold unrealistically high standards for their memory performance. Even minor forgetfulness is viewed as unacceptable and alarming, fostering a sense of cognitive failure (Pennington et al., 2015; Picon et al., 2022). This perfectionism may drive hypervigilant attentional styles, wherein patients monitor their memory processes excessively, paradoxically worsening their subjective experience of dysfunction (Ball et al., 2020; Teodoro et al., 2018).

Several cognitive-affective biases are commonly associated with FCD (Table 1):

- Impaired metacognitive ability: Patients with FCD often overestimate their cognitive deficits, even in the context of normal or near-normal neuropsychological test results. They may misattribute benign lapses to serious dysfunction, due in part to poor calibration between subjective experiences and objective performance (Ball et al., 2020; Bhome et al., 2022).
- Negative interpretation bias: Individuals with FCD may selectively attend to episodes of forgetfulness and interpret them as signs of progressive brain disease, reinforcing anxiety and worry (Bhome et al., 2022)
- Memory-related anxiety and societal expectations: Cultural narratives and personal beliefs about aging or family history of dementia can exacerbate memory-related fears. Many patients report assuming that cognitive decline is inevitable, thereby interpreting normal lapses as harbingers of irreversible decline (Teodoro et al., 2023).
- Cogniphobia: This term describes the avoidance of cognitively demanding situations due to fear of failure or embarrassment. Patients with FCD may reduce engagement in work or social settings, limiting opportunities to challenge maladaptive beliefs and perpetuating functional impairment (Ball et al., 2020; Cabreira et al., 2023a).

**Table 1:** Psychological features and metacognitive distortions in functional cognitive disorder.

**Feature**	**Description**	**Clinical relevance**
Impaired metacognition	Difficulty accurately monitoring or evaluating cognitive performance.	Leads to overestimation of deficits despite intact objective testing.
Memory perfectionism	Unrealistically high standards for memory functioning.	Fosters hypervigilance and misinterpretation of benign lapses as pathological.
Negative interpretation bias	Tendency to selectively attend to and catastrophize normal forgetfulness.	Reinforces anxiety and distress about cognitive health.
Memory-related anxiety	Fear of inevitable decline due to aging or genetic predisposition.	Increases symptom salience and promotes maladaptive beliefs.
Cogniphobia	Avoidance of cognitively demanding activities.	Reduces exposure to corrective experiences, reinforcing dysfunctional beliefs.
Psychiatric comorbidity	Co-occurrence of anxiety, depression, or trauma history.	May perpetuate or exacerbate cognitive complaints but does not account for FCD alone.
Impaired metacognition	Difficulty accurately monitoring or evaluating cognitive performance.	Leads to overestimation of deficits despite intact objective testing.
Memory perfectionism	Unrealistically high standards for memory functioning.	Fosters hypervigilance and misinterpretation of benign lapses as pathological.
Negative interpretation bias	Tendency to selectively attend to and catastrophize normal forgetfulness.	Reinforces anxiety and distress about cognitive health.
Memory-related anxiety	Fear of inevitable decline due to aging or genetic predisposition.	Increases symptom salience and promotes maladaptive beliefs.
Cogniphobia	Avoidance of cognitively demanding activities.	Reduces exposure to corrective experiences, reinforcing dysfunctional beliefs.

These cognitive and emotional dynamics create a self-perpetuating cycle in which excessive self-focus, distress, and avoidance reinforce the salience and persistence of cognitive complaints. Encouragingly, targeted psychological interventions such as CBT, which aim to address metacognitive beliefs, reduce avoidance, and promote adaptive thinking, have shown promising outcomes in this population (Cabreira et al., 2023a; Bhome et al., 2022).

It is important to distinguish metacognitive deficits from insight: while patients with FCD may exhibit metacognitive interference affecting their task performance, they can still retain good insight, often providing detailed, coherent accounts of their perceived cognitive difficulties (Wolstencroft, 2024; Larner, 2021). Mood and anxiety disorders are commonly comorbid with FCD. Although they may intensify cognitive concerns, FCD should not be viewed merely as a manifestation of affective disturbance. Rather, it constitutes a distinct clinical entity that overlaps with but is not reducible to depression or generalized anxiety disorder (Cabreira et al., 2023a; McWhirter et al., 2020). The presence of psychiatric comorbidities should be regarded as diagnostically informative, helping clinicians contextualize symptom development and persistence.

Ultimately, understanding the psychological architecture of FCD provides both explanatory insight and therapeutic direction. Shifting the clinical narrative away from structural pathology and toward dysfunctional self-appraisal, maladaptive illness beliefs, and metacognitive dysregulation allows clinicians to offer patients a hopeful and scientifically grounded explanation of their symptoms.

## 6 Long-term outcomes

Understanding the long-term trajectory of FCD is crucial for differentiating it from progressive neurodegenerative conditions, including MCI and dementia. One of the most common concerns among patients diagnosed with FCD is the fear that their cognitive symptoms represent the early stages of an irreversible decline. This anxiety is often intensified by diagnostic uncertainty and the overlap in early clinical presentations with neurodegenerative syndromes (Ball et al., 2020; Cabreira et al., 2023a). Long-term follow-up studies indicate that individuals with FCD typically maintain preserved daily functioning and independence over months to years, with objective performance remaining within normal limits. In a Brazilian tertiary memory clinic with low-education patients, around one-third of referrals were diagnosed with FCD, who also showed preserved cognitive performance despite higher subjective complaints (Pennington et al., 2019; Borelli et al., 2022; Schwilk et al., 2021).

It is important to note that FCD is defined by subjective cognitive complaints without consistent neurological signs (Cabreira et al., 2023a). Minor variations in neuropsychological test results may occasionally occur but do not correspond to specific neuroanatomical patterns and remain within normal limits. This feature distinguishes FCD from both SCD and neurodegenerative disorders.

However, longitudinal studies increasingly support the view that FCD is generally a stable, non-progressive condition, with most individuals maintaining consistent cognitive functioning over time (Bhome et al., 2022; McWhirter et al., 2020). One of the most compelling studies in this domain is a 10-year prospective follow-up involving individuals with SCD. Within this cohort, 31 out of 41 patients (~76%) who met criteria for FCD based on the Schmidtke inventory did not progress to MCI or dementia throughout the follow-up period (Cabreira et al., 2023a; Schmidtke and Metternich, 2009). This suggests that the use of structured, symptom-based criteria such as the Schmidtke scale may offer strong predictive validity in identifying individuals at low risk for neurodegeneration.

By contrast, dementia and mild cognitive impairment are characterized by a progressive trajectory, with cognitive decline that correlates with neuroimaging or biomarker changes and produces consistent impairments across domains. Whereas, individuals with FCD typically maintain preserved daily functioning and show intraindividual variability without progression, patients with dementia demonstrate a steady deterioration of memory, executive function, and independence (Ball et al., 2021). Importantly, anosognosia, reduced awareness of deficits, is common in dementia, while individuals with FCD tend to exhibit heightened symptom awareness and distress (Larner, 2021). These prognostic distinctions reinforce the clinical utility of recognizing FCD as a non-degenerative condition (Cabreira et al., 2023b).

These findings underscore the clinical value of making a positive diagnosis of FCD, rather than one based on exclusion. When clearly communicated, such a diagnosis can provide significant reassurance to patients, shift the therapeutic focus away from unnecessary diagnostic testing, and redirect care toward more appropriate psychological and rehabilitative strategies, such as cognitive-behavioral therapy or metacognitive training (Ball et al., 2020; Bhome et al., 2022).

Nevertheless, FCD is not a monolithic condition. Heterogeneity exists in clinical presentations and outcomes. While many individuals demonstrate cognitive stability or spontaneous improvement, others may experience persistent distress, often linked to unresolved psychological factors, comorbid anxiety or depression, or entrenched maladaptive beliefs about cognitive functioning (Pennington et al., 2015; Picon et al., 2022). In most cases, symptoms initially attributed to FCD remain stable, with no progression to neurodegenerative disorders observed over the follow-up period. In the cohort studied by Wakefield et al. (2018), none of the 20 FMD patients progressed to MCI or dementia, highlighting the typically benign and non-progressive course of the disorder. Only in the wider literature have rare cases been reported, which may reflect diagnostic uncertainty or true comorbidity (Ball et al., 2020; Wakefield et al., 2018).

Recent models conceptualize FCD as existing along a spectrum, ranging from brief, reversible episodes of cognitive dysfunction to more chronic and impairing functional syndromes (Ball et al., 2020; Teodoro et al., 2018). Crucially, this variability in presentation does not imply inevitable progression, but rather highlights the importance of individualized, longitudinal care and periodic reassessment when new clinical features emerge.

In practice, communicating the typically benign prognosis of FCD can be transformative for patients. It not only alleviates anxiety but also promotes better engagement with psychological therapies and supportive interventions. Moreover, it prevents premature entry into dementia care pathways, thus reducing unnecessary medicalization and reinforcing the functional nature of the condition (Cabreira et al., 2023a; McWhirter et al., 2020).

## 7 Assessment methods

**Figure 2:**
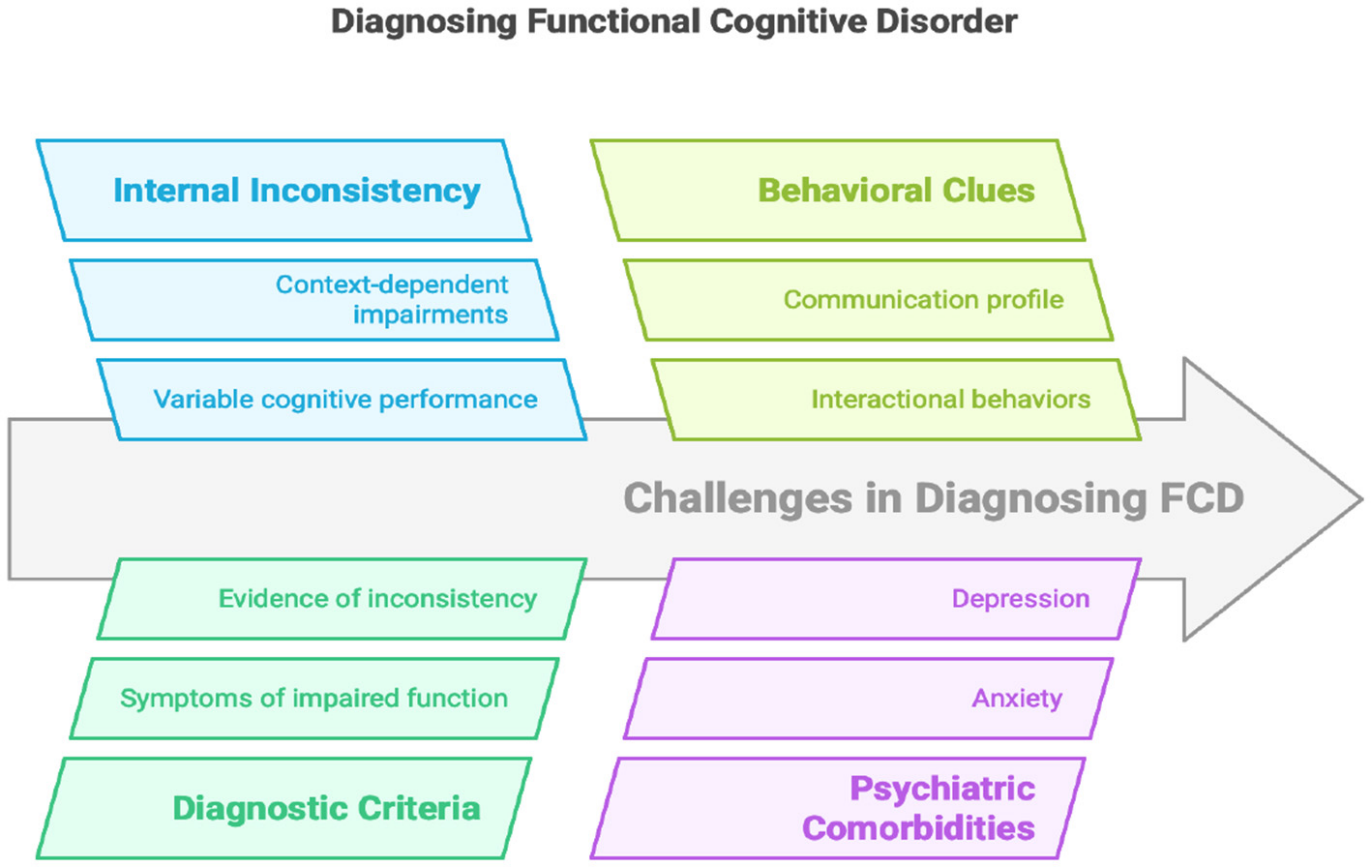
Key concepts in understanding functional cognitive disorder.

Diagnosing FCD requires a multimodal clinical approach that combines careful interviewing, observational data, formal neuropsychological testing, and validated structured tools (Figure 2). Unlike neurodegenerative disorders, where imaging, cerebrospinal fluid biomarkers, or genetic testing may support the diagnosis, FCD remains a clinical diagnosis, centered on the identification of positive features and the exclusion of progressive pathology. Positive features include internal inconsistency in cognitive performance, preserved functional independence, distinctive communication and interactional behaviors, and metacognitive markers such as memory perfectionism or heightened self-monitoring (Ball et al., 2020; Cabreira et al., 2023a; Bhome et al., 2022; Larner, 2021; Teodoro et al., 2023).

Various assessment methods contribute uniquely to the diagnostic process of FCD, as outlined in Table 2. These include neuropsychological testing to evaluate cognitive domains, brief cognitive screening instruments, medical symptom validity tests, structure self-report questionnaires, interactional and conversational assessments, structured psychiatric interviews the MINI, and standardized symptom-based frameworks like the Schmidtke Criteria. Together, these approaches provide complementary information that strengthens diagnostic confidence in functional cognitive presentations.

**Table 2:** Assessment tools and diagnostic aids in functional cognitive disorder.

**Assessment method**	**Purpose**	**Key diagnostic contribution**	**Limitation**	**References**
Neuropsychological testing	Evaluates cognitive domains (e.g., memory, attention).	Reveals normal or inconsistent impairments not aligning with neuroanatomical patterns; identifies red flags like discrepancy between subjective complaints and objective performance	May not capture subtle functional deficits; time-consuming	Ball et al., 2020; Wakefield et al., 2018; Zamarian et al., 2015
MoCA/MMSE	Brief cognitive screening tools.	Quick administration; identifies normal vs. abnormal performance; highlights discrepancy with subjective complaints	Limited specificity for FCD; may miss subtle cognitive fluctuations	Larner, 2014, 2005
Medical symptom validity tests	Tests response consistency and effort.	Helps identify attentional interference or metacognitive disruption rather than malingering.	Requires careful interpretation; may be affected by anxiety	Pennington et al., 2015; Picon et al., 2022
PHQ-15	Questionnaires assessing somatic symptoms, mood, sleep, and memory beliefs.	Elevated scores without objective deficits support functional diagnosis.	Does not assess cognition directly	Mascherek et al., 2011
HADS	Measures somatic	Captures bodily hypervigilance; identifies functional overlay	Does not assess cognition directly	Ball et al., 2020
PSQI	Evaluates sleep disturbances	Highlights contributing/perpetuating factors for cognitive complaints	Self-report; may not capture objective sleep quality	Tarik Elhadd, 2018
MMQ	Explores beliefs about memory and perceived control	Provides insight into dysfunctional metacognition; supports psychological formulation	Self-report; cultural differences may influence results	Pennington et al., 2015
Interactional and conversational assessment	Observation of communication patterns and symptom narratives.	Features like verbosity, coherence, and “attending alone” behavior support FCD.	Requires trained observer; subjective interpretation	Reuber et al., 2018; Teodoro et al., 2023
MINI interview	Structured psychiatric interview.	Identifies comorbid depression, anxiety, trauma; guides treatment planning	Time-consuming; requires trained	McWhirter et al., 2020
Schmidtke criteria	Symptom-based inventory.	Standardized framework; predictive validity for non-progression; supports structured clinical decision-making	Limited to memory-focused complaints; may not capture broader cognitive symptoms	Pennington et al., 2019; Schmidtke and Metternich, 2009

HADS, Hospital Anxiety and Depression Scale; FCD, Functional Cognitive Disorder; MMQ, Multifactorial Memory Questionnaire; MMSE, Mini-Mental State Examination; MoCA, Montreal Cognitive Assessment; PHQ-15, Patient Health Questionnaire-15; PSQI, Pittsburgh Sleep Quality Index.

### 7.1 Neuropsychological testing

Formal neuropsychological assessments are essential to evaluate cognitive domains such as memory, attention, language, and executive functioning. In FCD, testing often reveals normal or inconsistently impaired results, with patterns that do not conform to known neuroanatomical distributions (Ball et al., 2020; Wakefield et al., 2018). A typical red flag is a discrepancy between severe subjective complaints, defined as self-reported cognitive difficulties that significantly interfere with daily functioning or cause marked distress, and relatively intact objective performance in standardized neuropsychological tests (often within 0–1 standard deviation of normative values) (Ball et al., 2020; Pennington et al., 2015; Teodoro et al., 2023; Zamarian et al., 2015; Baker et al., 2018). Moreover, FCD patients may demonstrate intact performance on tasks requiring automatic processing, while underperforming on tasks perceived as cognitively demanding, highlighting the role of metacognitive interference.

### 7.2 Cognitive screening instruments

Brief cognitive screeners like the Montreal Cognitive Assessment (MoCA) and Mini-Mental State Examination (MMSE) are commonly used as initial tools. While not specific to FCD, normal performance on these tests, when paired with marked self-reported deficits, should raise clinical suspicion for a functional etiology (Larner, 2014, 2005). When such a discrepancy is present, clinicians should consider a functional etiology, especially in the absence of progressive decline.

### 7.3 Medical symptom validity tests (MSVT)

Performance validity tests such as the MSVT can help assess effort and test-taking consistency. In FCD, suboptimal performance is not necessarily indicative of malingering, but may instead reflect attentional disruption, anxiety, or metacognitive interference (Pennington et al., 2015; Picon et al., 2022). Interpretation requires clinical nuance and compassion, recognizing that metacognitive interference, rather than intentional deception, often underlies these results.

### 7.4 Structured questionnaires

Several validated self-report tools support the diagnosis of FCD and help assess common psychological comorbidities:

- Patient Health Questionnaire-15 (PHQ-15): Assesses the severity of somatic symptoms, often elevated in FCD due to bodily hypervigilance and health anxiety (Mascherek et al., 2011);
- Hospital Anxiety and Depression Scale (HADS): Screens for anxiety and depressive symptoms, which frequently co-occur with FCD and modulate symptom perception (Ball et al., 2020).
- Pittsburgh Sleep Quality Index (PSQI): Evaluates sleep disturbances, which are both risk factors and perpetuating elements in functional cognitive complaints (Tarik Elhadd, 2018);
- Multifactorial Memory Questionnaire (MMQ): Explores beliefs about memory and perceived control, providing insight into dysfunctional metacognitive frameworks common in FCD (Pennington et al., 2015);

High scores on these instruments, particularly in the absence of objective cognitive impairment, support the functional nature of the cognitive complaints and help identify psychological contributors to the patient's experience. Although these questionnaires assess somatic or affective symptoms rather than cognitive performance directly, they offer valuable context for understanding functional cognitive difficulties.

### 7.5 Interactional and conversational assessment

Patients' speech patterns and interpersonal behavior can be diagnostically informative. Tools developed by Reuber et al. (2018) and Teodoro et al. (2023) measure features such as narrative coherence, verbosity, and spontaneous detail, which help differentiate FCD from organic cognitive decline. For instance, patients with FCD often provide long, detailed accounts of their symptoms, demonstrating preserved verbal fluency and insight. To illustrate how these communication profiles manifest in clinical practice, consider the following brief examples (Cabreira et al., 2025):

Case 1: A 64-year-old retired woman presents alone to the clinic, reporting frequent forgetfulness, although she can recall information moments later. During the consultation, she provides coherent, detailed accounts of her memory lapses and brings written notes. Neurological examination and bedside cognitive testing are unremarkable. This case illustrates the application of FCD criteria: subjective cognitive complaints, preserved objective performance, associated anxiety, and characteristic communicative behaviors (Picon et al., 2022).

Case 2: A 44-year-old university teacher reports being easily distractible but now feels her memory is “worse than ever,” particularly during meetings. Colleagues have not noticed these difficulties. She attends the clinic alone. Neurological examination and bedside cognitive testing are unremarkable, with slightly slower immediate recall but preserved delayed recall. This vignette demonstrates the role of metacognitive interference and attentional dysregulation in functional cognitive complaints, highlighting how preserved objective performance can coexist with significant subjective impairment (Ball et al., 2020).

### 7.6 Mini International Neuropsychiatric Interview (MINI)

The MINI is a structured diagnostic interview designed to identify psychiatric comorbidities including mood, anxiety, and somatoform disorders. Given the high prevalence of such conditions in individuals with FCD, the MINI provides a valuable adjunct to cognitive testing. Identifying underlying or comorbid psychopathology is essential for effective treatment planning and for understanding the broader context of the patient's cognitive symptoms (Mavroudis et al., 2025).

### 7.7 The Schmidtke criteria

This 10-item inventory was designed to aid in diagnosing functional memory disorder by evaluating symptom inconsistency, preserved daily function, and distress. Its predictive validity for identifying patients unlikely to progress to dementia has been demonstrated in long-term follow-up studies (Cabreira et al., 2023a; Schmidtke and Metternich, 2009). Their use encourages a structured approach to differentiating functional memory complaints from early neurocognitive disorders and provides support for clinical decision-making.

## 8 Conclusion

Functional Cognitive Disorder represents an increasingly recognized but still underdiagnosed condition within the spectrum of cognitive disorders. Characterized by genuine cognitive complaints in the absence of progressive neurological pathology, FCD challenges traditional diagnostic paradigms, which often rely heavily on exclusion. However, emerging evidence highlights the presence of positive diagnostic features, such as internal inconsistency, distinct communication styles, and preserved interactional profiles, that support a confident clinical diagnosis.

Differentiating FCD from conditions like mild cognitive impairment, dementia, and pseudodementia is essential. Misclassification not only leads to inappropriate investigations and management strategies but can also heighten patient distress and reinforce maladaptive illness beliefs. In contrast, a timely and accurate diagnosis of FCD can provide significant reassurance and guide individuals toward evidence-based psychological interventions that target metacognitive dysfunction, memory-related anxiety, and associated behavioral avoidance.

Longitudinal data support the view of FCD as a non-progressive condition for most individuals, with stable cognitive trajectories over time. These findings, alongside tools such as the Schmidtke criteria and structured conversational profiling, offer clinicians robust strategies for identifying FCD in memory clinic populations.

Despite these advances, FCD remains an under-researched area. Further work is needed to refine diagnostic criteria, validate assessment tools, and develop tailored treatment pathways. Moreover, there is a pressing need for an international consensus on diagnostic criteria and the development of specific clinical guidelines, given the significant impact of accurate diagnosis on patient outcomes. Recognizing FCD as a legitimate and treatable clinical entity is essential for improving patient care, reducing stigma.
